# Die Anwendung der Virtuellen Realität in der Behandlung psychischer Störungen

**DOI:** 10.1007/s00115-022-01378-z

**Published:** 2022-09-02

**Authors:** N. Tsamitros, A. Beck, M. Sebold, M. Schouler-Ocak, F. Bermpohl, S. Gutwinski

**Affiliations:** 1 Psychiatrische Universitätsklinik der Charité – Universitätsmedizin Berlin im St. Hedwig-Krankenhaus/Institutsambulanz, Müllerstr. 56–58, 13349 Berlin, Deutschland; 2 Klinik für Psychiatrie und Psychotherapie, Campus Charité Mitte, Charité – Universitätsmedizin Berlin, corporate member of Freie Universität Berlin, Humboldt-Universität zu Berlin, and Berlin Institute of Health, Berlin, Deutschland; 3 Klinik für Psychiatrie und Psychotherapie, Campus Benjamin Franklin, Charité – Universitätsmedizin Berlin, corporate member of Freie Universität Berlin, Humboldt-Universität zu Berlin, and Berlin Institute of Health, Berlin, Deutschland; 4 Fakultät Gesundheit, Health and Medical University, Campus Potsdam, Potsdam, Deutschland

**Keywords:** Angststörungen, Digitale Psychiatrie, Expositionstherapie, Avatar, Immersion, Anxiety disorders, Digital psychiatry, Exposure therapy, Avatar, Immersion

## Abstract

**Hintergrund:**

Die Virtuelle Realität (VR) ermöglicht das Eintauchen in eine interaktive, digitale Welt mit realitätsnahen Erfahrungen, die im Rahmen therapeutischer Intervention kontrolliert und personalisiert eingesetzt werden können. In dieser Übersichtsarbeit werden die aktuellen Forschungsergebnisse zur VR in der Behandlung psychischer Störungen zusammengefasst.

**Methode:**

Selektive Literaturrecherche in PubMed und über Google Scholar.

**Ergebnisse:**

Eine zunehmende Anzahl von Publikationen beschreibt unterschiedliche Einsatzformen der VR in der Behandlung psychischer Störungen. Die Mehrheit der VR-Anwendungen basiert auf Adaptionen bereits etablierter psychotherapeutischer Methoden, insbesondere der Expositionstherapie. Die Virtuelle Expositionstherapie (VRET) in der Behandlung der spezifischen Phobie und der Agoraphobie mit Panikstörung ist laut metaanalytischen Daten gleich wirksam wie die traditionelle Expositionstherapie in vivo. VRET für die soziale Phobie ist signifikant wirksamer als Warte- oder Placebo-Kontrollgruppen, aber im Vergleich zur Expositionstherapie in vivo sind die metaanalytischen Befunde derzeit inkonsistent. VRET bei der posttraumatischen Belastungsstörung (PTBS) ist laut Metaanalysen gleich wirksam wie eine aktive Psychotherapie. Für die VR-basierte Behandlung der psychotischen Störungen gibt es positive Befunde bezogen auf Reduktion des Stimmenhörens. Bei Patienten mit einer Abhängigkeitserkrankung kann mittels VR „craving“ induziert werden mit noch unzureichend belegter diagnostischer und therapeutischer Relevanz.

**Schlussfolgerung:**

Die VRET kann als Erweiterung der psychotherapeutischen Behandlung der Angststörungen angeboten werden. Vielversprechende Befunde der VR-basierten Therapien der PTBS und der psychotischen Störungen implizieren den Bedarf weiterer Forschung zur Klärung ihrer Effektivität und Sicherheit. Im Bereich der Abhängigkeitserkrankungen ist die Evaluation klinisch orientierter VR-Anwendungen erforderlich.

## Abstract (en)

**Background:**

Virtual reality (VR) enables immersion in an interactive digital world with realistic experiences, that can be applied for controlled and personalized interventions. This review summarizes the current research on VR in the treatment of mental disorders.

**Methods:**

Selective literature search in PubMed and Google Scholar.

**Results:**

An increasing number of publications report the therapeutic application of VR for the treatment of mental disorders. Most VR applications are based on established therapy approaches, such as exposure therapy. According to meta-analytic data, virtual exposure therapy (VRET) for specific phobia and agoraphobia with panic disorder is as effective as traditional in vivo exposure therapy. VRET for the treatment of social phobia is significantly more effective than waitlist and placebo control groups with, however, currently inconsistent metanalytic results when compared to in vivo exposure therapy. VRET for the treatment of posttraumatic stress disorder (PTSD) is similar in effectiveness compared to active psychotherapy. For psychosis, positive results have been reported for the VR-based treatment of auditory verbal hallucinations. For patients with a substance use disorder, VR can induce craving, with still unverified diagnostic and therapeutic relevance.

**Conclusions:**

VRET can broaden the psychotherapy options for anxiety disorders. Encouraging results of VR-based treatments for psychosis and PTSD indicate the need for further research concerning its effectiveness and safety. In the field of substance use disorders, evaluation of clinical-orientated VR applications is needed.

## Hintergrund

Virtuelle Realität (VR) beschreibt eine Mensch-Maschine-Schnittstelle, die es dem Nutzer ermöglicht, in eine computergenerierte, dreidimensionale Umgebung einzutauchen und mit ihr zu interagieren ([6]; Tab. 1 und 2). Seit den ersten Einsätzen der VR in der Psychiatrie in den 1990er-Jahren (Angstreduktion bei Patienten mit Höhenangst) wurden VR-Anwendungen für ein breites Spektrum psychiatrischer Krankheitsbilder stetig weiterentwickelt und erforscht [33, 36]. Auch wenn Anwendungen in VR weitgehend noch nicht im klinischen Alltag integriert sind, wird ihre Wirksamkeit durch eine steigende Anzahl wissenschaftlicher Publikationen gestützt [9]. Für das Jahr 2005 fanden sich unter dem Stichwort *„virtual reality“* in der Medline-Datenbank 267 Publikationen sowie 11 Publikationen unter der Stichwortkombination *„virtual reality“* und *„psychiatry“*. Die gleichen Suchanfragen ergaben für das Jahr 2021 jeweils 2926 und 204 Publikationen.

**Table 1:** 

*Cave Automatic Virtual Environment (CAVE):*	Ein Raum zur Projektion dreidimensionaler Bilder an die Wandflächen. Der Nutzer trägt eine 3D-Brille und kann sich im Raum frei bewegen. Dadurch können Objekte gesehen werden, die in der Luft zu schweben scheinen, sodass eine Illusionswelt entsteht
*Head-Mounted Display (HMD):*	Ein auf dem Kopf zu tragendes, visuelles und akustisches Ausgabegerät, welches mit augennahen Bildschirmen und Lautsprecher ausgestattet ist. Durch einen „head tracker“ (Sensoren für Erfassung der Lage und Bewegung des Kopfs) können die Bilder durch die Position und Blickrichtung des Nutzers angepasst werden. HMDs zählen zu den meistverwendeten VR-Geräten

**Table 2:** 

*Immersion:*	Zustand eines „tiefen Eintauchens“ in eine virtuelle Realität sowie die Fähigkeit des VR-Systems, eine „lebendige Erfahrung“ zu generieren, welche den Nutzer von der physischen Realität entfernt
*Präsenz:*	Das subjektive Gefühl des „Dort-Seins“ während der Nutzung der VR
*Avatar:*	Eine grafische Darstellung des Nutzers in der VR-Welt

Ziel unserer Übersichtsarbeit ist eine Zusammenfassung der Forschungsergebnisse für den Zeitraum seit 2015 über die wichtigsten Anwendungsmöglichkeiten der VR in der Behandlung psychischer Störungen. Wir fokussieren uns in dieser Übersichtsarbeit auf Angststörungen, posttraumatische Belastungsstörung (PTBS), psychotische Störungen und Abhängigkeitsstörungen, da es sich hierbei um die robusteste Datenlage handelt und die innovativsten Studien durchgeführt wurden.

## Methode

Selektive Literaturrecherche in PubMed und über Google Scholar mit der Suchstrategie: (virtual OR virtual reality) AND (psychiatry OR psychotherapy OR anxiety OR fear OR depression OR phobia OR PTBS OR traumatic OR psychosis OR schizophrenia OR addiction OR alcohol). Besonders berücksichtigt wurden systematische Reviews, Metaanalysen und Leitlinien für den Publikationszeitraum zwischen Januar 2015 und August 2021.

## Angststörungen

Die Expositionstherapie wird als Erstlinientherapie der spezifischen Phobie und in Kombination mit anderen Elementen auch bei weiteren Angststörungen und der PTBS von den deutschen und internationalen Leitlinien empfohlen [2, 21, 39]. Trotz der klaren Indikation wird die Expositionstherapie bisher eher seltener in der Patientenversorgung eingesetzt, was unter anderem in praktischen Hindernissen und einem ungünstigen Aufwand-Vergütungsverhältnis begründet liegt [34]. Ähnlich wie die Exposition in vivo oder in sensu (imaginativ) kann die Exposition durch Simulation der phobischen Stimuli in der VR Angst und PTBS-Symptome bei den Betroffenen auslösen [30]. Beispielsweise konnten Angst- und Stressreaktionen während einer Exposition in der VR durch Veränderungen der elektrodermalen Aktivität gemessen werden [44].

Mehrere Metaanalysen haben die Effektivität der virtuellen Expositionstherapie (VRET) in der Behandlung der sozialen Phobie, der Agoraphobie mit Panikstörung sowie der spezifischen Phobie untersucht [7, 23, 32, 43]. Die VRET zeigte sich in diesen Studien für die genannten Angststörungen in der Wirksamkeit vergleichbar mit der Expositionstherapie in vivo sowie signifikant wirksamer als Kontrollgruppen bestehend aus regulärer Behandlung, psychologischen Placebo-Interventionen oder Wartelisten [7, 15, 43]. In der Metaanalyse von Wechsler et al. (*n* = 371) zeigte sich im Prä-Post-Vergleich VRET und Expositionstherapie in vivo große Effektstärke (Hedges g = 1,00 und 1,07) [43]. Als einzige Ausnahme fanden sich inkonsistente Befunde für die soziale Phobie im Vergleich zur Expositionstherapie in vivo. [23, 32, 43]. Die Evidenz der VRET zu Agoraphobie mit Panikstörung ist eingeschränkt, da sie sich auf lediglich drei Studien bezieht. Nur eine Subgruppe der Patienten hatte eine Panikstörung ohne Agoraphobie, sodass hierzu keine Schlussfolgerung gezogen werden konnte [7, 43]. Es konnte keine VRET-Studie für die generalisierte Angststörung gefunden werden, die die Inklusionskriterien der o. g. Metaanalysen erfüllte [7, 43]. Die Autoren der Metaanalysen berichteten noch, dass die Effektivität der VRET zwischen den einzelnen Studien variierte, und schlussfolgerten, dass weitere Forschung zur Identifikation der Wirkprinzipien der VRET notwendig sei [23, 43].

**Figure 1:**
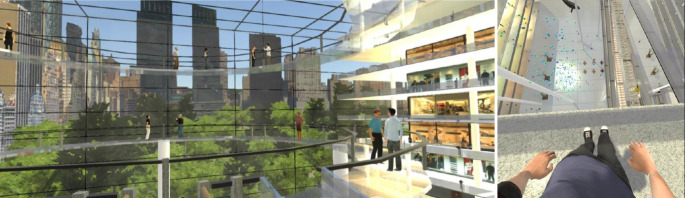

In der Gesamtbetrachtung bezüglich VRET-Studien für Angststörungen zeigt sich, dass Therapeuten während der Sitzungen eine aktive Rolle übernehmen. Im Gegensatz dazu untersuchten Freeman et al. eine automatisierte VR-Anwendung in einer randomisierten Einfachblindstudie hinsichtlich der Effektivität auf Höhenangst im Vergleich zu einer Kontrollgruppe mit „üblicher“ bzw. keiner Behandlung (Abb. 1; [17]). Es zeigte sich sowohl nach sechs Therapiesitzungen als auch zum Follow-up (vier Wochen) eine signifikante Verbesserung im Vergleich zur Kontrollgruppe. In einer Netzwerkmetaanalyse mit über 17 Studien und 946 Teilnehmern mit Höhenangst von Chou et al. war die automatisierte VR-Anwendung von Freeman et al. auf dem ersten Platz bezüglich ihrer Effektivität bei der Angstreduktion unter 19 unterschiedlichen Therapiemethoden für die Behandlung der Höhenangst. Miloff et al. verglichen ebenfalls automatisierte VRET mit integrierten digitalen Spielelementen mit Exposition in vivo in einer randomisierten Studie für Patienten mit Arachnophobie (*n* = 100) in einer Nicht-Inferioritäts-Studie [31]. Die Nicht-Inferiorität konnte erst in dem Drei- und Zwölf-Monats-Follow-up erreicht werden. Direkt nach der Intervention war VRET der Exposition in vivo unterlegen.

Laut einer Metaanalyse von Benbow und Anderson über 46 Studien mit insgesamt 1056 Teilnehmern für die Behandlung von Angststörungen war die Abbruchrate mit 16 % unter VRET geringfügig kleiner als bei der Exposition in vivo und bei der kognitiven Verhaltenstherapie (KVT) der Angststörungen (19,6 %. laut einer Metaanalyse von Fernandez et al.) [4, 14]. Unter den Gründen für den vorzeitigen Abbruch der Behandlung wurde für VRET an erster Stelle angegeben, dass die VRET als nicht immersiv genug erlebt wurde. Bei der Exposition in vivo stellte die Angst vor dem phobischen Stimulus den häufigsten Abbruchgrund dar [4]. Lindner et al. formulierten die „Lowered Threshold Hypothesis“, nach welcher VRET die Schwelle für Patienten reduziert, Konfrontationen mit phobischen Situation im realen Leben einzugehen [29]. In Patienten mit Arachnophobie zeigte sich, dass eine starke Symptomreduktion nach der VRET-Sitzung in der Nachuntersuchungszeit zu mehr aktiv geplanten Expositionen und so zu einer stärkeren weiteren Symptomreduktion führte [29].

Die langfristigen Effekte der VRET, zumindest bei der Behandlung der Aviophobie (Flugangst), werden durch eine retrospektive Studie von Gottlieb et al. unterstützt [20]. Die Autoren befragten 98 Patienten, die in den letzten drei Jahren mit VRET behandelt wurden. Diese berichteten von einer signifikant größeren Anzahl an Flugreisezeiten im Vergleich zur Zeit vor der VRET-Behandlung.

Laut S3-Leitlinie zur Behandlung der Angststörungen kann VRET zur Behandlung der sozialen Phobie als Begleittherapie zusätzlich zu einer Standardpsychotherapie angeboten werden, sollte diese aber nicht ersetzen [2]. Zusätzlich kann bei Spinnen‑, Höhen- oder Flugphobie VRET eingesetzt werden, wenn die traditionelle Exposition in vivo nicht verfügbar oder nicht möglich ist [2]. Für die Panikstörung und Agoraphobie gibt es laut Autoren der Leitlinien keine ausreichende Evidenz, und die VRET sollte daher nicht angeboten werden [2].

## Posttraumatische Belastungsstörung

Für die Behandlung der PTBS werden derzeit drei VR-basierte Therapieinterventionen beschrieben [25]. Die meistuntersuchte VR-Intervention ist die VRET für PTBS, die aus der prolongierten Exposition weiterentwickelt wurde und einem ähnlichen Vorgehen wie die VRET bei den Angststörungen folgt [13, 25]. Statt einer „traditionellen“ Exposition in sensu (imaginativ) werden Patienten mithilfe eines HMD in einer virtuellen Umgebung mit traumaspezifischen Stimuli konfrontiert mit dem Ziel der Angstreduktion durch Habituation [25]. Eine weitere Therapiemethode, die „Multi-Modular Motion-Assisted Memory Desensitization and Reconsolidation Therapy“ (3MDR), basiert auf der „Eye Movement Desensitization and Reprocessing Therapy“ (EMDR) unter Berücksichtigung der Reduktion von Vermeidungsstrategien seitens der Patienten [13, 25]. Im Rahmen der 3MDR befindet sich der Patient in einem CAVE-Raum (Cave Automatic Virtual Environment) und läuft auf einer Laufbahn zu projizierten traumaspezifischen Bildern, während EMDR-Therapieelemente eingesetzt werden [13, 25]. Die Wirksamkeit dieser Verfahren wird mit konsistenten Befunden mehrerer Metaanalysen beschrieben [7, 12, 13]. In der aktuellsten Metaanalyse von Eshuis et al. über neun VRET- und zwei 3MDR-Studien mit insgesamt 438 Patienten erwiesen sich die VR-Interventionen als wirksamer als die inaktiven Kontrollgruppen (standardisierte Mittelwertdifferenz zur Nachbehandlung −0,64, 95 % CI −1,05 bis −0,22) [13]. Darüber hinaus erwiesen sie sich als gleich wirksam wie die etablierten traumafokussierten und anderen Psychotherapien hinsichtlich der Intensität der PTBS-Symptome (standardisierte Mittelwertdifferenz zur Nachbehandlung −0,25, 95 % CI −0,77 bis 0,27) [13]. Die Autoren betonen, dass aufgrund der heterogenen Ergebnisse der Studien, der relativ hohen Abbruchraten von 21,9 % und der nicht ausreichenden Evaluation der Nebenwirkungen und Sicherheitsaspekte weitere Studien erforderlich sind, damit zukünftig VRET als alternative Therapiemethode für Patienten mit PTBS angeboten werden kann [13]. Eine dritte Therapieintervention ist die „Action-Centered Exposure Therapy“ (ACET), die eine aktive Interaktion mit der traumatischen Umgebung im Gegensatz zum VRET ermöglicht, z. B. das Fahren eines Lastkraftwagens für Patienten mit Verkehrstrauma, um neue Lernprozesse zu aktivieren [25]. Hier fehlen trotz positiver Ergebnisse bezüglich Reduktion der PTBS-Symptomatik aus zwei Fallstudien noch randomisierte kontrollierte Studien [25]. Bisher wird VR nur in den Leitlinien der Internationalen Gesellschaft für Traumatischen Stress als eine Methode mit zunehmender Evidenz eingestuft [5].

Insgesamt werden VR-Interventionen für die posttraumatische Belastungsstörung entwickelt und erforscht, sind aber für die Integration in die Routineversorung noch nicht ausgereift genug.

## Psychotische Störungen

Die Wirksamkeit der VR-basierten KVT für Patienten mit Verfolgungswahn und sozialer Vermeidung im Rahmen einer psychotischen Störung im Vergleich zur Warteliste wurde in einer randomisierten kontrollierten einfach verblindeten Studie (*n* = 58) untersucht [35]. Hierbei wurden die Patienten unter therapeutischer Begleitung ermutigt, paranoide Ideen zu hinterfragen, Vermeidungsverhalten (wie Vermeidung von Blickkontakt) zu überwinden und negative Erwartungen zu überprüfen. Kurzzeitige Angst sowie paranoide Gedanken waren in der Nachuntersuchung im Vergleich zu vor Behandlungsbeginn sowie zu Patienten der Warteliste signifikant reduziert. Allerdings zeigte sich die Gesamtdauer der sozialen Interaktionen der Patienten im „Echtleben“ unverändert. Freeman et al. verglichen in einer randomisierten Studie die Effektivität einer einmaligen 30-minütigen VR-basierten KVT-Sitzung mit einer VR-Exposition bei Patienten (*n* = 30) mit Psychose und Verfolgungswahn [16]. Im Vergleich zur VR-Exposition konnte durch die VR-basierte KVT eine deutliche Reduktion des Verfolgungswahns und der Anspannung in realen Situationen erreicht werden.

Bei VR-Avatar-Therapien erstellen Behandler gemeinsam mit Patienten mit einer psychotischen Störung und akustischen Halluzinationen eine digitale grafische Darstellung der Person, welche den Vorstellungen der Patienten über die Person, aus der die Stimmen kommen, entspricht [10, 11, 28, 41]. Während wiederholter Therapiesitzungen lernt der Patient mithilfe des Therapeuten besser mit den Stimmen umzugehen [10, 28, 41]. Die ersten drei Studien von Leff et al., Craig et al. und du Sert et al. berichteten von einem positiven Effekt der Avatar-Therapie bezüglich der Häufigkeit und Intensität des Stimmenhörens im Vergleich zur regulären oder supportiven Behandlung [10, 28, 41]. Aali et al. analysierten die o. g. drei Studien mit insgesamt 195 Teilnehmern und hinterfragten die klinische Bedeutung der relativ kleinen Effekte, der fehlenden binären Outcomes, diskutierten diese im Rahmen eines Bias-Risikos und empfahlen weitere unabhängige Studien [1].

## Abhängigkeitserkrankungen

Craving („Suchtdruck“) und die Reaktivität auf substanzbezogene Stimuli als zentrale Elemente von Konsumrückfällen sind Bestandteile der Forschung, der Diagnostik und Therapie von Abhängigkeitserkrankungen [3, 22, 24]. Die VR-Exposition mit suchtassoziierten Stimuli löste in mehreren Studien Craving bei Patienten mit Nikotin‑, Alkohol-, sowie Methamphetaminabhängigkeit aus, wobei zum Teil olfaktorische Reize zur Verstärkung eingesetzt wurden [40]. Die Wiederholung von Expositionen führte in mehreren Studien zu einer signifikanten Craving-Reduktion [27, 42]. Bisher fehlen aber randomisierte kontrollierte Studien zur Wirksamkeit der VR-Anwendungen bezüglich Verbesserung von Abstinenzraten oder -dauern [27, 42].

## Nebenwirkungen und Risiken

Die am häufigsten erfasste Nebenwirkung der VR-Anwendung ist der „cybersickness“, welche der klassischen Seekrankheit ähnelt [26]. Das Risiko für solche Beschwerden ist von den technologischen Eigenschaften der VR-Geräte, vom Inhalt (z. B. statisch vs. Bewegung) und von individuellen Faktoren abhängig [37, 38].

## Schlussfolgerungen

VR ist noch nicht Teil der Routine-Patientenversorgung, obwohl das Potenzial für die Diagnostik und Therapie im Bereich der Psychiatrie und Psychotherapie seit den ersten klinischen Studien in den 1990er-Jahren wiederholt belegt wurde. Im Bereich der Angststörungen gibt es für die VRET der spezifischen Phobie ausreichend metaanalytische Daten, die ihren Einsatz im klinischen Alltag rechtfertigen und mittlerweile Eingang in Leitlinien gefunden haben [2]. Für die soziale Phobie kann VRET basierend auf der aktuellen Evidenzlage als Ergänzung einer Psychotherapie angeboten werden [7, 43]. Für die Agoraphobie mit Panikstörung ist die Evidenz zur VRET wegen der geringen Anzahl der Studien eingeschränkt [7, 23, 32, 43]. Während Metaanalysen den Nutzen von VRET im Rahmen der PTBS-Behandlung im allgemeinen unterstützen, fehlen noch Studien, die zwischen verschiedenen Traumatypen differenzieren [12, 13]. Obwohl VRET der traditionellen Exposition in vivo nicht überlegen ist, erscheint VRET aufgrund ihrer leichteren Durchführbarkeit und der Patientenpräferenz als Erweiterung der Therapiemöglichkeiten sinnvoll [8, 19]. In der Behandlung von Patienten mit einer psychotischen Störung gibt es vor allem positive Daten für persistierendes Stimmenhören. Hierzu sind multizentrische unabhängige randomisierte kontrollierte Studien für die Überprüfung der Therapiekonzepte erforderlich [1, 35, 41]. Für den Einsatz der VR-Exposition gegenüber substanzassoziierten Stimuli in klinischen Therapieprogrammen der Abhängigkeitserkrankungen fehlen noch randomisierte kontrollierte Studien über klinische Outcomes, z. B. Rückfallraten [27].

Die Mehrheit der VR-Anwendungen erfordern eine Steuerung durch Therapeuten. Dagegen wurden automatisierte VR-Therapien mit standardisierter Anleitung nur vereinzelt angewendet [17, 18, 31]. Bisherige VR-Anwendungen bestehen überwiegend aus Adaptionen bereits etablierter Therapieinterventionen. Die Identifikation und Optimierung der Wirkprinzipien der VR-Anwendungen sowie die Entwicklung von für das neue Medium spezifischen VR-Anwendungen könnte deren Potenzial nutzen, innovative Therapiekonzepte zu ermöglichen.

### Infobox Auswahl Anbieter deutschsprachigen VR-Therapien für Angststörungen

**Invirto**, https://invirto.de/, kontakt@invirto.de

**Neomento**, https://neomento.de/, hello@neomento.de

**VR Coach**, https://www.vr-coach.at/, info@vr-coach.at

**VTplus**, https://www.vtplus.eu/, kontakt@vtplus.eu

## Fazit für die Praxis

- Die Virtuelle Realität (VR) ermöglicht die Simulation von realitätsnahen Situationen, womit die Durchführung der Expositionstherapie vereinfacht wird.
- Die VR-Expositionstherapie (VRET) beweist eine gute Wirksamkeit bei der Behandlung der Angststörungen, insbesondere der spezifischen Phobien, und kann als Alternative zu der traditionellen Expositionstherapie in vivo bei entsprechender Präferenz der Patienten eingesetzt werden.
- Innovative VR-basierte Therapien für die posttraumatische Belastungsstörung (PTBS), psychotische Störungen und die Abhängigkeitserkrankungen werden intensiv erforscht.
- Die häufigste Nebenwirkung der VR ist „cybersickness“.
